# Balancing the Virulence and Antimicrobial Resistance in VISA DAP-R CA-MRSA Superbug

**DOI:** 10.3390/antibiotics11091159

**Published:** 2022-08-27

**Authors:** Rossella Salemi, Alessandra Zega, Elvira Aguglia, Flavia Lo Verde, Giuseppe Pigola, Stefania Stefani, Viviana Cafiso

**Affiliations:** Department of Biomedical and Biotechnological Sciences, University of Catania, 95123 Catania, Italy

**Keywords:** VISA, CA-MRSA, omics, virulence, antimicrobial resistance

## Abstract

Background: Methicillin-resistant *Staphylococcus aureus* (MRSA) with intermediate resistance to Vancomycin (VISA) is reported worldwide. These strains frequently emerge among hospital-associated (HA)-MRSA and rarely within community-acquired (CA)-MRSA. Here, the genomic and transcriptomic adaptations distinguishing VISA daptomycin resistant (DAP-R) CA-MRSA, which emerged in a hospitalized patient under glycopeptide treatment, were explored. Methods: Whole-genome sequencing, RNA-Seq and bioinformatics were carried out. Results: Our CA-MRSA clustered in the USA400 lineage showing additional antimicrobial resistance (AMR) versus DAP and glycopeptides. Resistomics revealed adaptations related to glycopeptide, daptomycin and rifampin resistance (*mpr*F nsSNPS and overexpression of glycopeptide and daptomycin-resistance related genes). Similar changes were detected in virulence traits (*agr*A HI-nsSNPs and toxin gene underexpression), in which a decrease was observed despite the abundance of virulence-related genes. Our results predicted a balance in adaptations, decreasing the virulence and biological costs to support the co-occurrence of extensive AMR in a hypervirulent genomic background. Conclusion: Our data show that VISA DAP-R CA-MRSA shifts the potential hypervirulent behavior of CA-MRSA towards the acquisition and maintenance of extensive AMR, by a decrease in virulence and biological costs mediated by a “compensatory modulatory mutation” silencing the Agr quorum-sensing cascade.

## 1. Introduction

Hospital- and community-acquired methicillin-resistant *Staphylococcus aureus* (MRSA) are considered high-priority micro-organisms because the severity of their infections is often difficult to treat [1]. Exploiting a plethora of complex infection-related mechanisms of infection, *S. aureus* causes a wide range of serious infections, i.e., abscesses of various organs, bacteremia, infective endocarditis (IE), pneumonia, as well as osteoarticular, skin and soft tissue, pleuropulmonary and device-related infections. Complicated therapeutical management is often necessary to treat MRSA infections, even with the emergence of new Gram-positive drugs [1]. From an evolutionary viewpoint, *S. aureus* constantly has reprogrammed its antimicrobial resistance (AMR) mechanisms in response to increasing antimicrobial pressure and virulence mechanisms to adapt its host defense response. Antimicrobial and immune system activity are key factors in conditioning bacterial survival [2,3,4,5,6,7] and in the selection of new lineages or pathogen variants. Both mechanisms are crucial for survival under adverse conditions such as host immune system defenses, antimicrobial treatment and the need to survive in new challenging niches. 

Reflecting these two evolutionary and selective drives, MRSA selected three different variants related to the modality of transmission: (i) healthcare-associated methicillin-resistant *S. aureus* (HA-MRSA), (ii) community-acquired *S. aureus* (CA-MRSA) and (iii) livestock-associated *S. aureus* (LA-MRSA), each of which is strongly adapted to different environments. Among these, HA-MRSA infections are mainly reported following contact with healthcare settings. Community-associated MRSA (CA-MRSA) infections have been reported since the 1990s in individuals who had no prior hospitalization [8], whilst LA-MRSA are associated with livestock [9]. CA-MRSA is particularly virulent compared to HA-MRSA in that, on the contrary, it is highly resistant to antimicrobial treatment [10,11]. This aspect reflects different genomics of HA-MRSA versus CA-MRSA. HA-MRSA is characterized by a huge pool of antimicrobial resistance (AMR) genes and lower virulence-related genes. CA-MRSA has a lower content of AMR genes (in general restricted to β-lactams and macrolides) and a higher pool of virulence-related genes, including the Panton-Valentine Leukocidin (PVL) toxin gene. This is a β-pore-forming cytotoxin creating pores in the membranes of infected cells that can cause necrotic lesions in the skin, in the mucosa and can also determine necrotic hemorrhagic pneumonia [10,11].

Virulence and resistance factors share similar mechanisms of dissemination and co-selection between species or genera. These mechanisms are mediated by horizontal gene transfer (HGT) of mobile genetic elements (MGEs), compensatory or adaptive mutations [12,13] and intrinsic factors related to the nature of micro-organisms such as biofilm-producing or intracellular bacteria [2,3,6,7], involvement of the same cellular structures such as efflux pumps [4], porins [5], cell wall alterations [6] and two-component regulatory systems [7].

MRSA antimicrobial resistance towards first-choice and last resort antimicrobials such as glycopeptides and daptomycin is one of the great challenges for the current management of MRSA infections. These strains emerge typically and frequently among HA-MRSA because of the selective pressure arising during severe antimicrobial treatment; on the other hand, they are very rare among CA-MRSA [14,15].

In our experimental plan, global integrated genomic and transcriptomic profiling was applied to better understand the adaptations of a new VISA DAP-R CA-MRSA superbug that attracted our attention for its biological characteristic of extensive antimicrobial resistance, including glycopeptide and daptomycin resistance.

## 2. Results

A comparative genomic and transcriptomic approach was exploited to uncover the gained adaptations by VISA DAP-R CA-MRSA, acquired under the selective pressure of teicoplanin treatment, versus its VSSA DAP-S CA-MRSA isogenic parents. 

First, the genomic lineage and background characterizing VISA DAP-R CA-MRSA, as well as the genomic markers associated with the take-over of extensive antimicrobial resistance (VISA and DAP-R), were determined. In-depth genomic characterization (phylogeny, genomic epidemiology, molecular typing, whole-genome non-synonymous single nucleotide polymorphisms) was conducted by whole-genome sequencing (WGS) and bioinformatics, focusing mainly on the traits impacting AMR and Virulence.

Similarly, a pool of the statistically significant differentially expressed genes (DEGs), their expression trend (over- or under-expression) and the main enriched dysregulated KEGG pathways were determined by RNA-seq and bioinformatics. 

Finally, the phenotypical biofilm production ability (together with already known delta-hemolysis production ability) and growth-curve kinetics were evaluated to assess the production of prototype (adhesion and toxigenicity, respectively) virulence factors and the biological AMR and virulence fitness costs.

### 2.1. Biological Fitness Costs of AMR and Virulence

AMR and virulence biological fitness costs were evaluated in terms of variation in generation time and lag growth-phase duration in 1-R vs 1-S, as previously published [16]. A longer generation time was found in 1-R (60′) than in 1-S (30′). In addition, growth kinetic experiments showed that the lag growth-phase of VISA DAP-R CA-MRSA 1-R was greater (2.00 h) than VSSA DAP-S CA-MRSA 1-S (1.0 h), as well as more similar to MW2 CA-MRSA (2.30 h) and *S. aureus* ATCC 29213 (2.25 h) (Table 1).

### 2.2. Biofilm Production

Biofilm production assays showed that 1-S and 1-R MRSA strains were not biofilm producers (Table 2).

### 2.3. Phylogeny 

**Figure 1:**
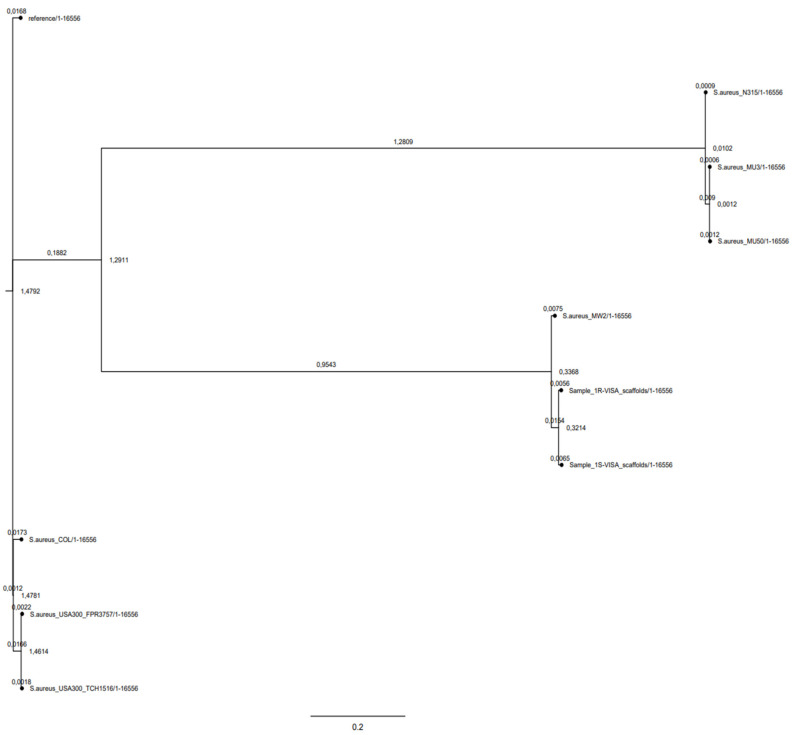
Phylogenetic SNP tree of 1-S and 1-R versus a representative group of HA-MRSA (Reference:SA-NCTC8325, SA-COL, SA-Mu3,SA-Mu50,SA-N315), and CA-MRSA (SA-USA-300 FPR3757, SA-USA-300TCH 1516, SA-MW2 USA-400).

CSI phylogeny clearly showed the clusterization of 1-S and 1-R MRSA strains in the USA-400 CA-MRSA phylogenetic lineage. The phylogenetic tree showed a close relationship between the 1-S/R MRSA strain pair and the ST-1 MRSA cluster. This highlighted the strong phylogenetic and genomic relationship with the MW2 CA-MRSA, notoriously a USA400, *agr*-III, CA-MRSA prototype (Figure 1). 

### 2.4. Genomic Characterization 

The WGS characterization confirmed and increased our previously published data [17], i.e., ST-1, SCCmec-type IVa, *agr*-type III, spa-type t127, genomic integration of two staphylococcal phages and five plasmid replication initiating genes (Table 3). 

Resistomics, consistently with the antibiotypes, showed the following acquired AMR-genes: (1) *ant(6)-Ia* and *aph(3′)-III* (aminoglycoside-resistance); (2) *bla*Z and *mec*A (β-lactam-resistance); (3) *erm*C (macrolide-resistance); (4) *tet*K (tetracycline-resistance) in both strains (Table 3). 

Virulome analysis found the major staphylococcal virulence factors including 22 adherence related genes, 22 immune evasion related genes, 8 secretion system coding genes, 14 exoenzyme encoding genes and 26 toxin coding genes, including *luk*S/F encoding the PVL in both strains (Table 3).

### 2.5. Genomic SNPs 

SNPomes, mapped on MW2 CA-MRSA RefGen, evidenced 619 and 609 wgSNPs in the 1-S and 1-R genomes versus MW2 RefGen, respectively, whilst 104 wgSNPs were recorded between 1-S and 1-R. Furthermore, in 1-R versus 1-S, HI-nsSNPs were found in four genes, among which *agr*A (Arg170*) and 22 MI-nsSNPs “already associated to AMR” in *mpr*F (Thr345Ala) were related to DAP-R and r*po*B nsSNPs for RIF-R (His481Tyr). In addition, MI-nsSNPs in *cap*8H (Tyr130His), *cap*8K (Val120Gly), *eta* (Leu47Ile), *sdr*D (Thr1313Ser) and *ebh* (Val1768Asp) virulence-related genes were found in 1-R versus 1-S (Table 3, Table 4 and Table 5).

### 2.6. Comparative Transcriptomics 

A complete overview of comparative transcriptomes in 1-R vs. 1-S revealed 145 overexpressed and 265 under-expressed transcripts. Among these, applying the medium filter and EASE score threshold ≤ 1.0, David enrichment analysis evidenced two main enriched clusters of DEGs, including the KEGG pathways, i.e., glycolysis and gluconeogenesis, microbial metabolism in diverse environments, mismatch repair, homologous recombination and DNA replication, as shown in Table 6. 

Filtered TS and SI library-integrated output data of the DEGs showed interesting dysregulation in subsets of genes related to AMR resistance and virulence (adhesion and toxigenicity). Extrapolating the transcriptomic traits impacting the biology of CA-MRSA DAP-R VISA phylogenomic lineage, key differential expression was found in the following GO-TERM biological process (BP) gene-clusters: (1) AMR-related traits: overexpression in tcaA (Glycopeptide-resistance), murF (D-cycloserine), dltA and mprF (DAP/VAN-R); underexpression in norA and norB (FQs) and in tet38 (Tetracycline-resistance); (2) Virulence-related traits: Adhesion: under-expression in sdrC and sdrD (ser-Asp rich proteins), eap/map (extracellular adherence protein/MHC analogous protein); Effector delivery system: underexpression in esaA (Type VII secretion system); Exotoxins: underexpression in hla, hld, hlgB/C (hemolysins), seh (enterotoxin H) and in the enterotoxin homologous type A coding gene MW1552; Membrane-acting toxin and Superantigen: underexpression in spa (staphylococcal protein A); Exoenzymes: underexpression in lip (Lipase) and splB (serine-protease); Immune modulatory proteins: overexpression in cap8N (capsule), sbi (staphylococcal binder of immunoglobulin); underexpression in cap8C/G/P (capsule), isaB (immunodominant staphylococcal antigen B; Biofilm: underexpression in icaA (intercellular adhesin A); (3) AMR and virulence transcriptional regulators: overexpression in vraRS, sigB, saeR and rot; underexpression in walKR, srrA, agrBCA, sarS, graR and sarS (Table 7).

**Figure 2:**
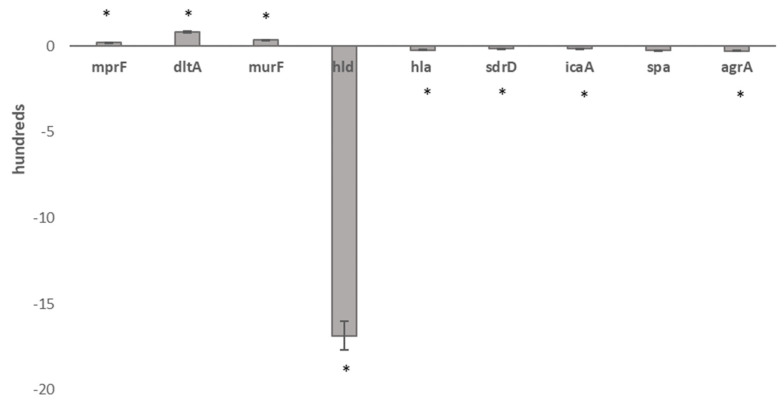
Real-time qPCR validation of RNA-seq data in 1-R vs. 1-S. Legend: * Statistically significant differentially expressed genes.

Real-time qPCRs of the set of characterizing transcripts, murF, hld, hla, dltA, mprF, spa, agrA, icaA and sdrD, clearly confirm the expression trends found in RNA-seq transcriptional data outputs (Figure 2).

## 3. Discussion

HA-MRSA and CA-MRSA emerged as different variants of clear adaptive events that occurred within the same species. The genomic composition of the two MRSA lineages clearly indicated that evolution led to highly balanced genomes with HA-MRSA strongly adapted to survive in extreme antimicrobial selective pressure whilst CA-MRSA overcame the host-response [18,19,20,21].

In this scenario, our data describe, for the first time, a new CA-MRSA superbug that emerged as a result of a convergent cross-selection mechanism that makes the CA-MRSA superbug able to decrease its hypervirulence, supporting and maintaining extensive antimicrobial resistance. 

These new genomically related USA-400, ST-1, spatype-t127, *agr*-III, SCC*mec*IVa, PVL-positive, glycopeptide and daptomycin-resistant CA-MRSA were characterized by the co-occurrence of an extensively antimicrobial resistance profile and decreased virulence despite their great virulence potential.

New insights arose on the layout of genomic and transcriptomic adaptations occurring in this exceptional circumstance in which MRSA harbors simultaneous adaptations related to and impacting antimicrobial resistance and virulence. 

Two convergent key motifs emerged as key points of its success: (i) the selection of a compensatory “Silencing regulatory” mutation in the agr-locus, strategic quorum sensing regulator pathway, involved in virulence and antibiotic resistance; (ii) dysregulation in essential primary metabolic pathways associated with a prevalent underexpression trend in DEGs. These co-events determine and allow a balancing of the antimicrobial resistance and virulence adaptations that lead to the setup of increased antimicrobial resistance by decreasing virulence and restoring intrinsic biological costs.

Focusing on antimicrobial-resistance adaptive traits, our findings showed features related to the acquired resistance towards glycopeptides and daptomycin. Resistomics highlighted that MI-nsSNPs in *mpr*F are associated with glycopeptide and daptomycin resistance. In addition, AMR-related transcriptomics revealed overexpression in t*ca*A, associated with reduced glycopeptide resistance, as well as in *dlt*A and *mpr*F related to daptomycin-glycopeptide resistance. Looking uniquely at glycopeptide resistance, our data indicate that *tca*A can have an impact on the level of teicoplanin resistance in terms of MIC values rather than on teicoplanin resistance phenotype acquisition. This observation could seem in contrast with previous findings reporting that *tca*A inactivation and *tca*RAB deletion determine an increase in teicoplanin resistance [22,23]. These data speculatively implicate that if *tca*RAB inactivation increased teicoplanin resistance, on the contrary, an overexpression should decrease teicoplanin resistance. Our data show *tca*A overexpression in the teicoplanin-resistant 1-R (TEC MIC 32 mg/L) versus its teicoplanin-susceptible parent 1-S, indicating a positive association with increased teicoplanin resistance. Therefore, the role of *tca*A genes in teicoplanin resistance is still not fully clarified. 

In *S. aureus*, daptomycin resistance and reduced susceptibility to vancomycin are often associated with multifactorial mechanisms [24,25,26,27]. Our data confirm *dlt*A and *mpr*F overexpression as the basis of a mechanism of electrostatic repulsion responsible for daptomycin resistance in MRSA, as previously published [24,25,26,27]. *dlt*A and *mpr*f determine an alteration of the surface envelope by alanylation and lysinylation teichoic acids as well as in the membrane phosphatidylglycerol, responsible for increased positive net charge that blocks DAP docking to its target [24,26]. From these new RNA-seq experiments, *dlt*A and *mpr*F overexpression were clearly, statistically significant and differentially expressed in 1-R with respect to 1-S. These data contrasted with our previous findings, likely due to the different methodology used [17]. 

Furthermore, in VISA DAP-R CA-MRSA, other traits associated, as expected, with other AMR-resistance traits were found. These were acquired resistant SNPs related to rifampin resistance (*rpo*B), and dysregulation in *mur*F related to D-cycloserine resistance, in n*or*A and *nor*B implicated in FQ-resistance, and *tet*38 involved in tetracycline-resistance. 

Focusing on virulence, virulomics revealed the characteristic huge virulence potential of VISA DAP-R CA-MRSA due to a great pool of virulence-related genes conferring potentially extraordinary abilities of adherence, anti-phagocytosis, immune system evasion and toxigenicity in agreement with previously published data [28]. 

However, new consideration arises from our data regarding the presence of a stop codon compensatory modulatory mutation in *agr*A. This allows the CA-MRSA genomic background to shift towards a wide AMR phenotype. The agrA compensatory regulatory mutation, associated or not to the dysregulation in the master regulators (vraRS, sigB, saeR, rot overexpression and walKR, srrA, agrBCA, sarS, graR underexpression), confers the ability to drastically decrease CA-MRSA toxigenicity to adapt the strain to support the antimicrobial resistance burden needed for maintaining extensive AMR. A stop codon, as a “compensatory regulatory mutation” leading to a 170 AA residue truncated AgrA, acts with a “double smart strategy”. The former is as an “agr quorum-sensing silencer”, the latter is a “virulence-modulator”. It is well known that *agr*-locus positively regulates the production of numerous toxin-coding genes by antisense regulatory RNA-III and negative adhesion [29]. AgrA is the histidine kinase receptor of the agr-system involved in the regulation of *S. aureus* virulence in response to bacterial cell density. Truncated AgrA is crucial and strategic to block the cascade leading to the activation/repression of the regulatory pathways related both to toxigenicity and antimicrobial resistance. Virulence-related transcriptomic data confirmed that AgrA non-functionality is concomitant with the underexpression of the agr-locus, hemolysin coding genes (*hla*,*hld*,*hlg*B/C) and enterotoxins (*seh*, MW1552), and is further supported by the lack of delta-hemolysin production ability. 

Moreover, these data confirmed other results that clearly demonstrate that VISA or DAP-R MRSA is typically associated with the decreased agr-functionality frequently showed in *agr*-II MRSA with increased and extensive AMR [17,24,25,26,27].

Focusing on biological fitness costs, resistant bacteria pay biological fitness costs for their changes that can diminish their growth rate; our data could appear in contrast with this observation as our VISA DAP-R CA-MRSA showed different strategies to balance the AMR fitness costs [16,30]. VISA DAP-R CA-MRSA faces AMR fitness costs by increasing the generation time compensated with an extension of the lag growth phase that restores the growth kinetics of more susceptible *S. aureus*. Additionally, two strategies of transcriptomic adaptations were evidenced. VISA DAP-R CA-MRSA was able to balance the biological costs shifting the transcriptional rate as follows: (i) an underexpression trend in differentially expressed genes, including numerous accessory virulence-related genes, i.e., toxin genes (as discussed above), adhesin genes (sdrC, sdrD, eap/map), type VII secretion system genes (esaA), membrane-acting toxin and superantigen genes (spa), exoenzyme genes (lip, splB), immune-modulatory genes coding for the capsular antigens (cap8C/G/P), immunodominant staphylococcal antigen B (isaB) and biofilm production (icaA), confirmed by the lack of biofilm production; (ii) dysregulation of several essential primary metabolic pathways (glycolysis, gluconeogenesis, microbial metabolism in diverse environments, mismatch repair, homologous recombination and DNA replication) and master transcriptional regulators (overexpression in vraRS, sigB, saeR and rot and underexpression in walKR, srrA, agrBCA, sarS and graR).

## 4. Conclusions

Our data reveal that convergent evolution, exerted by environmental selective pressure, selects MRSA hybrid variants with extensive antimicrobial resistance towards the last resort antimicrobials, i.e., glycopeptides and daptomycin, within “decreased hypervirulent” CA-MRSA by a balanced pool of adaptations supporting and maintaining the burden of enhanced antimicrobial resistance. 

This mechanism is very interesting and to be taken into consideration as it could determine the selection of new hybrid variants that are “extremely dangerous” in the potential severity of infections and in the therapeutical options that could dramatically complicate the future scenario of bacterial infection management.

## 5. Materials and Methods

### 5.1. Bacterial Strains

One *S. aureus* isogenic strain-pair (1-S and 1-R) of ST-1, *agr*-III, delta-hemolysin negative, was recovered from a patient hospitalized in an Italian hospital, as previously described [17]. Briefly, a 69-year-old female patient was admitted on 30th of March 2012 to the coronary care unit (CCU) with fever and cardiac decompensation due to mitralic and aortic valve insufficiency. Echocardiography revealed infective endocarditis (IE) on the native mitral and biological aortic valves, and the 1-S MRSA was isolated from blood cultures. Treatment with intravenous (IV) teicoplanin plus gentamycin was begun with initial defervescence. On the 11th day of treatment, the patient was again febrile, and blood cultures yielded a second MRSA (1-R strain) with daptomycin and vancomycin resistance. Vancomycin and gentamycin were then suspended, and IV quinupristin/dalfopristin (Q/D) was prescribed. However, because of the initial unavailability of this drug, IV linezolid was administered for 10 days. Later, Q/D was again available and administered alone for 4 more weeks. During the latter treatment, blood cultures were negative, and the patient was afebrile. However, at the end of this antibiotic treatment, the patient developed a central venous catheter (CVC)-related infection due to *Stenotrophomonas maltophilia* and had fatal septic shock, as previously described [17]. In detail, the first (1-S) MRSA strain was susceptible to vancomycin, teicoplanin, daptomycin, linezolid, trimethoprim/sulfamethoxazole and rifampin but resistant to cefoxitin (CFX), ampicillin, amoxicillin/clavulanate, fluoroquinolones, erythromycin, clindamycin and tetracycline. On the contrary, the second isogenic MRSA (1-R), isolated under teicoplanin therapy, was characterized by additional resistance to glycopeptides and daptomycin according to the EUCAST guidelines 2021 [31]. The antimicrobial susceptibility and molecular characterization were in part, previously characterized [17] and reported in Table 2 and Table 3.

### 5.2. Biological Costs Determined by the Growth Kinetics Test

The 1-S and 1-R MRSA strains were tested for maximum growth rate and length of the lag growth phase. Both MRSA strains were inoculated in Mannitol Salt Agar (MSA) (Oxoid) plates and incubated overnight at 37 °C. One colony of each isolate was resuspended in phosphate-buffered saline and diluted to an optical density of 0.05 at 600 nm. In total, 1 mL of each suspension was then added to 50 mL of BHI broth. Optical density readings at 600 nm were taken every 30 min over 6 h and plotted against time. The generation time was considered the time it takes for bacterial population duplication, and the lag phase duration was at the beginning of the maximum growth rate. Generation time and lag growth phase duration for 1-S and 1-R were compared by the independent samples t-test, and a *p*-value ≤ 0.05 was considered the cut-off of statistical significance. This experiment was repeated three times, and CA-MRSA MW2 and *S. aureus* ATCC29213 were used as controls [32].

### 5.3. Biofilm Production

1-S and 1-R MRSA strains were tested for their ability to produce biofilm by a spectrophotometric quantitative assay. Each strain was grown in Tryptone Soy Broth (Oxoid, Basingstoke, UK), with the addition of 0.25% glucose (TSBG), and biofilm production assays were performed in microtiter plates as previously described [33].

### 5.4. Whole-Genome Sequencing

Genomic DNA was extracted using the PureLink Genomic DNA Mini Kit (Invitrogen, Waltham, MA, USA) following the manufacturer’s protocol. DNA quality was evaluated by Qubit, and its concentration was determined by Picogreen (Life Technologies, Carlsbad, CA, USA). Whole-genome sequencing (WGS) was performed using the Illumina Mi-Seq sequencing system, using both a paired-end library with 150 bp reads (400 bp average insert size) and a mate-pair library with 250 bp reads (8 kb average insert size). After the sequence data generation, raw reads were processed by Fast QC (v0.11.7) to assess data quality, and the Trimmomatic tool (v0.38) was used to remove sequencing adapters for paired end reads to filter low-quality bases (Q_score < 30) and short reads (<150 bp) as well as for mate pair reads to the process by requiring a minimum base quality of 20 (Phred scale) and a minimum read length of 100 nucleotides to filter out sequences composed only by Ns and to improve the per base score of the mate pair reads. The total number of PE and MP reads is reported with the estimated coverage in Supplementary Table S1. The trimmed reads were used for downstream analysis [27,34].

### 5.5. De-Novo Genome Assembly

De novo genome assembly was performed using SPAdes software (v3.12.0). Reads were initially normalized with khmer 1.3, and then they were error-corrected using the Bayesian Hammer utility of SPAdes. Finally, assembly was performed using the recommended parameters for such Illumina data. The SPAdes software produced a contigs file for each sample, post-assembly controls and metrics were evaluated using Quast software (v4.6.3) and are presented in Supplementary Table S2 [27,34].

### 5.6. Gene Annotation

The assembled contigs were processed using Prokka software (1.14.6) to predict genes and annotate those sequences using a core set of conserved prokaryotic genes [27,34].

### 5.7. Single Nucleotide Variants (SNVs)

For SNVs, genomic re-sequencing was performed from the paired-end library raw reads as already published [27,34]. Briefly, Illumina raw reads were trimmed by the Trimmomatic tool (v0.38), requiring a minimum base quality of 20 (Phred scale) and a minimum read length of 36 nucleotides. Only trimmed reads were included in the downstream analysis. Each sample was aligned by BWA v. 0.7.5 on *S. aureus* MW2 (BA000033.2) and used as a reference genome. Each .bam file was sorted by Samtools (v.0.1.19), and duplicate reads were marked using the Picard Mark Duplicates utility. Complex variants, SNVs and indels were detected by “Freebayes” (v.0.9.14), requiring a minimum mapping quality of 25 (Phred scale) and a minimum base quality of 30. 

Sequenced reads were properly aligned with the reference genome, 97.28% for 1-S and 97.29% for 1-R.

To select only SNVs present in the VISA DAP-R- CA-MRSA, wg nsSNVs were computationally filtered versus those present in the VSSA DAP-S CA-MRSA parent. All non-synonymous SNPs present in COL-R isolates were confirmed by Sanger sequencing.

### 5.8. Whole-Genome Single Nucleotide Polymorphisms (wgSNPs) Effect Prediction

wgSNPs effect prediction was evaluated using snpEff (v.4.3T). High (HI), low (LI), moderate (MI) or modifier impact (MFI) was assigned according to the criteria previously published [35]. In detail: high impact: the variant is assumed to have disruptive impact, probably causing protein truncation, loss of function or triggering nonsense-mediated decay; low impact: assumed to be mostly harmless or unlikely to change protein behavior; moderate impact: a non-disruptive variant that might change protein effectiveness; modifier impact: usually non-coding variants or variants affecting non-coding genes where predictions are difficult, or there is no evidence of impact [34]. Only high (HI) and moderate (MI) effect wgSNPs were considered and, consequently, described.

### 5.9. Phylogeny and Genomic Epidemiology 

The whole-genome sequencing raw data were evaluated by free tools of the Center for Genomic Epidemiology (CGE, http://www.genomicepidemiology.org/, accessed on 1 July 2022) to investigate the genetic and molecular features of the strain-pair. In detail, spaTyper (v1.0) was used to determine the staphylococcal protein A (*spa*) type of each strain, SCCmecFinder (v1.2) identified the staphylococcal cassette chromosome mec (SCCmec), PlasmidFinder (v2.0) was used for plasmid search, PHAge Search Tool (PHAST) was used considering only the prophage regions detected with a completeness score >90 to detect the prophages, VirulenceFinder (v2.0) was used to identify the virulence factors to define the Virulome and ResFinder (v3.2) was used for the detection of the acquired antimicrobial resistance genes [36,37,38,39,40,41,42]. Analyses were performed with default setting parameters. 

### 5.10. RNA-Seq

#### 5.10.1. RNA-Seq Bacterial Cultures

An aliquot of an overnight culture was diluted 1:50 in 30 mL of brain heart infusion (BHI) in a sterile 50 mL flask (OD_600_ nm 0.05) to obtain approximately 5 × 10^5^ CFU/mL inoculum for each strain. Cells were grown under shaking at 250 rpm with normal atmospheric conditions at 37 °C and harvested in the exponential growth phase (OD_600_ 0.5, 2 × 10^8^ CFU/mL ∼ 3–4 h). RNA extraction started immediately after cell harvesting to maintain RNA integrity. The cell density was determined by colony counting after plating onto Mueller–Hinton (MH) agar and incubation.

#### 5.10.2. RNA-Seq Libraries

RNA-seq was carried out using the Illumina Mi-seq sequencing platform. To improve RNA-seq data, two replicates using two different libraries were conducted, a Single-End Library with 50 bp reads (SI, Short-Insert Library) and a Paired-end Read Library with 150 bp reads (TS, Tru-Seq Library) and an average insert size of 350/400 bp. 

#### 5.10.3. RNA-Extraction

Specific RNA extractions for the Tru-Seq Library and Short-Insert Library preparation were performed according to the specific protocols, as a strategy to optimize the collected RNA-seq data, as previously published [27,34].

#### 5.10.4. Tru-Seq Library Preparation

The total RNA quality was verified using a 2200 TapeStation RNA Screen Tape device (Agilent, Santa Clara, CA, United States), and its concentration was ascertained using an ND-1000 spectrophotometer (NanoDrop, Wilmington, DE, United States). The Agilent TapeStation 2200 system, an automated instrument for nucleic acid gel electrophoresis, assigned RNA integrity number (RIN) values ranging from 1 to 10, with 10 being the highest quality. Only samples with preserved 16S and 23S peaks and RIN values > 8 were used for the library’s construction. The RIN values > 8 indicate intact and high-quality RNA samples for downstream applications, as previously published [27,34]. Ribosomal RNA was removed using the Bacteria Ribo-Zero rRNA Removal Kit from 2 μg of RNA. The depleted RNA was used for the Illumina Truseq RNA stranded kit without PolyA enrichment. The obtained libraries were evaluated with high-sensitivity D1000 screen Tape (Agilent Tape Station 2200), and the indexed libraries quantified with the ABI9700 qPCR instrument using the KAPA Library Quantification Kit in triplicates was according to the manufacturer’s protocol (Kapa Biosystems, Woburn, MA, United States). From the pooled library, 2 nm final concentrations were used for sequencing with a 150 PE read sequencing module [27,34].

#### 5.10.5. Short-Insert Library Preparation 

After ribosomal depletion, sequencing libraries were created using the Illumina mRNA-seq sample preparation kit following the supplier’s instructions, except that total RNA was not fragmented, and double-stranded cDNA was size-selected (100–400 bp) to maximize the recovery of small-size RNA. The prepared libraries were valued with high-sensitivity D1000 screen Tape (Agilent Tape Station 2200), as described for the TS Library. The indexed libraries were quantified in triplicate with the ABI7900 qPCR instrument using the KAPA Library Quantification Kit, according to the manufacturer’s protocol (Kapa Biosystems, Woburn, MA, United States). From the pooled library, 5 μL at a final concentration of 4 nM were utilized for MiSeq sequencing with an A single-end stranded library with reads of a 50-bp sequencing module [27,34].

#### 5.10.6. Tru-Seq Library Raw Read Post-Processing 

After sequence data generation, raw reads were processed using FastQC (v.0.11.2) to assess data quality. The sequenced reads were then trimmed using Trimmomatic (v.0.33.2) to remove only sequencing adapters for PE reads. A minimum base quality of 15 (Phred scale) over a four-base sliding window was required. Only sequences with a length above 36 nucleotides were included in the downstream analysis, and likewise, only trimmed reads were included in the downstream analysis [27,34].

#### 5.10.7. Short-Insert Library Raw Read Post-Processing 

After sequence data generation, raw reads were processed using FastQC (v.0.11.2) to assess data quality. Reads were then trimmed using Trimmomatic (v.0.33.2) to remove sequencing adapters for single-end reads, requiring a minimum base quality of 15 (Phred scale) and a minimum read length of 15 nucleotides. Only trimmed reads were included in the downstream analysis [27,34].

#### 5.10.8. Tru-Seq and Short-Insert Read Analysis

TS and SI RNA-seq reads were annotated on *S. aureus* MW2 (BA000033.2) used as RefGen, as well as transcripts assembled and quantified using Rockhopper (v.2.03) [27,34]. Analyses were run using default parameter settings with verbose output to obtain expression data. Rockhopper normalizes read counts for each sample using the upper quartile gene expression level. Starting from the *p*-values calculated according to the Anders and Huber approach, differentially expressed genes (DEGs) were assigned by computing q-values ≤ 0.01 based on the Benjamini–Hochberg correction with a false discovery rate of <1%. In addition, Rockhopper is a versatile tool using biological replicates when available and surrogate replicates when biological replicates for two different conditions are unavailable, considering the two conditions under investigation as surrogate replicates for each other [27,34].

### 5.11. DAVID Enrichment Analysis

The online tool DAVID (v.6.8) (http://david.abcc.ncifcrf.gov/ accessed on 1 July 2022) was used to detect affected pathways among DEGs. The gene lists of the strain-pair, grouped according to over- and underexpressed genes, were uploaded as Official Gene Symbols of the *S. aureus* MW2 reference genome, automatically selecting the list type (Gene list) of *S. aureus*. The Functional Annotation Chart was obtained using an EASE score threshold ≤0.5 and a minimum count number of four genes. The DAVID Functional Categories were investigated by the KEGG pathway and PANTHER Classification System [43] and grouped in annotation clusters refined of the same genes.

### 5.12. Real-Time qPCR validation

To validate RNA-seq data, real-time qPCRs for a set of characterizing transcripts, *mur*F, *dlt*A, *mpr*F, *hld*, *hla*, *spa*, *agr*A, *ica*A, and *sdr*D, were carried out in the same RNA-seq growth phase. Primer lists are provided in Tab.S3; *gyr*B was used as a normalizer gene. Real-time qPCRs and statistical analyses were carried out as previously published [31,32].

### 5.13. DNA-Sequencing and RNA-Sequencing Data Accession Number

The genomic and transcriptomic reads were deposited in the National Center for Biotechnology Information (NCBI) Genome database in the Sequence Read Archive (SRA) under the BioProject PRJNA860577 with the Biosample Genomic Paired End and RNA-seq raw sequences n° SAMN29849119, SAMN29849120 and with Biosample Genomic mate-pair draft sequences n°SAMN30428603, SAMN30428604.

## Figures and Tables

**Table 1:** Generation times and lag growth phase kinetics.

Strain	Sccmec Cassette	Agr-Group	DAP and Glycopeptide Resistance	PVL	Generation Time Average (min)	Lag Growth Phase Duration Average (h)
1S CA-MRSA	IVa	III	VSSA, DAP-S	+	30	1.0
1R CA-MRSA	Iva	III	VISA, DAP-R	+	60	2.00
MW2 CA-MRSA	Iva	III	VSSA, DAP-S	+	30	2.30
*S. aureus* ATCC 29213	II	II	VSSA, DAP-S	-	30	2.25

**Table 2:** Antimicrobial patterns, δ-hemolysis and biofilm production.

Strains	MIC					Biofilm	δ-hemolysis
	CFX	DAP	VAN	TEC	RIF		
1-S	32	0.5	1	1	<0.06	No Producer	Negative
1-R	48	2	8	32	>256	No Producer	Negative

MICs and δ-hemolysis data were previously published in Capone et al., 2016.

**Table 3:** Genomic, resistomic and virulomic characterization.

Strain	MLST	PFGE	Agr-Type	Spa-Type	SCCmec	Plasmids	Phages	Virulome		Resistome	Resistance Gene SNPs			
											Gene	NUCL. Change	AA Change	AMR
1-S	ST-1 [17]	USA 400 [17]	III [17]	t127	IVa	rep5a (pN315) rep7a (repC-Cassette) rep7c (MSSA476) rep10 (pDLK1) rep16 (pSAS)	Phage Staph phiJB-NC028669 Phage Staph-ST398_4NC023499	*ebpS, ebh, vwb, eap, atl, fib, spa, clfA, clfB vwb, fnbA, fnbB, eap, sdrC, sdrD, sdrE sdrH, icaRABC, cna*	Adherence	Aminoglycosides-R: ant(6)-Ia, aph(3′)-III β-lactams-R: blaZ, mecA Macrolides-R: ermC Tetracyclines-R: tetK	gyrA	251C > T	S84L	FQs
											grlA	239 C > T	S80F	FQs
								hla, hlgACB, hld [17], hlb, eta, lukE, lukSF-PV, set2, set3, set16, set17, set18, set19 set20, set21, set22, set23 set25, set26, SEnt-ike, SEntHSEntA, sal, tst	Toxins					
								sspABC, geh, nuc hysA, lip, splABCF sspB2, sak, scn	Exoenzyme genes					
								cap8J, cap8I, cap8H, cap8G, capF, cap8E, capD, cap8C, cap8B, cap5A, isb, capD, cap8P, capC, capB,capA, cap8O, cap8M, cap8M, cap8L, cap8K, cap5M	Host Immune Evasion					
								essB, esaB, essA esaA, esxA, esxB, esaC, essC	Secretion System					
1-R	ST-1 [17]	USA 400 [17]	III [17]	t127	IVa	rep5a (pN315) rep7a (repC-Cassette) rep7c (MSSA476) rep10 (pDLK1) rep16 (pSAS)	Phage Staph phiJB-NC028669 Phage Staph-ST398_4NC023499	*ebpS, ebh, vwb, eap, atl, fib, spa, clfA, clfB vwb, fnbA, fnbB, eap, sdrC, sdrD, sdrE sdrH, icaRABC, cna*	Adherence	Aminoglycosides-R: ant(6)-Ia, aph(3′)-III β-lactams-R: blaZ, mecA Macrolides-R: ermC Tetracyclines-R: tetK	gyrA	251C > T	S84L	FQs
											grlA	239 C > T	S80F	FQs
											rpoB	1441C > T	H481Y	RIF [17]
											mprF	1033A > G	T345A	DAP [17]
								hla, hlgACB, hld [17], hlb, eta, lukE, lukSF-PV, set2, set3, set16, set17, set18, set19 set20, set21, set22, set23 set25, set26, SEnt-ike, SEntHSEntA, sal, tst	Toxins					
								sspABC, geh, nuc hysA, lip, splABCF sspB2, sak, scn	Exoenzyme genes					
								*cap8J, cap8I, cap8H, cap8G, capF, cap8E, capD* *cap8C, cap8B, cap5A, isb, capD* *cap8P, capC, capB, capA, cap8O, cap8M, cap8M, cap8L* *cap8K, cap5M*	Host Immune Evasion					
								essB, esaB, essA esaA, esxA, esxB, esaC, essC	Secretion System					

**Legend:** [17] n° of the reference.

**Table 4:** HI nsSNPs in 1-R vs. 1-S on MW2 CA-MRSA RefGen mapping.

Gene	Product	nsSNPs
HI-nsSNPs		
MW1125	YfhO family protein	Gly75 *
MW1482	proline dipeptidase	Thr265Ala
MW2347	8-amino-7-oxononanoate synthase (*bio*F)	Glu272 *
MW1963	Accessory gene regulator protein A (*agr*A)	Arg170 *

Legend: * stop codon.

**Table 5:** MI nsSNPs in 1-R vs. 1-S on MW2 CA-MRSA RefGen mapping.

Gene	Product	nsSNPs
**MI- nsSNPs**		
MW0014	Cyclic-di-AMP phosphodiesterase	Ile186Met
MW0165	N-acetylmuramic acid 6-phosphate etherase	Ile156Asn
MW0447	Ribonuclease M5	Asp98Glu
MW1054	Exfoliative toxin A	Leu47Ile
MW1080	Pseudouridine synthase	Glu294Val
MW1664	Peroxiredoxin osmotic stress-related protein	Asn87Lys
MW1826	DUF2154 domain-containing protein VraT	Ala59Glu
MW2107	D-ornithine--citrate ligase SfnaD	Leu195Trp
MW2286	Malate:quinone oxidoreductase 1	Val280Glu
MW2393	D-histidine (S)-2-aminobutanoyltransferase CntL	Glu31Leu
MW2533	HTH-type transcriptional regulator	Gly41Asp
MW0131	Capsular polysaccharide synthesis enzyme H	Tyr130Hys
MW0134	Capsular polysaccharide synthesis enzyme K	Val120Glu
MW2070	Cobalt-zinc-cadmium resistance protein czcD	Asp52Glu
MW1283	Dihydrodipicolinate synthase DapA	Ala101Thr
MW1247	Phosphatidylglycerol lysyl-transferase MprF	Thr345Ala Leu538Phe
MW2304	Proton/sodium-glutamate symport protein	Val232Glu
MW1324	Extracellular matrix-binding protein Ebh	Val1768Asp
MW2287	L-lactate permease LctP	Ile178Phe
MW1307	UDP-NAG--NAM-(pentapeptide) pyrophosphoryl-undecaprenol N-acetylglucosamine transferase MurG	Ile121ASn
MW0497	DNA-directed RNA polymerase subunit beta RpoB	His481Tyr
MW0517	Serine-aspartate repeat-containing protein SdrD	Thr1313Ser

**Table 6:** DAVID enrichment analysis of the transcriptome DEGs in 1-R vs. 1-S.

Kegg Pathway	Gene Number in Cluster	Products	*p*-Value
Annotation Cluster 1 Enrichment Score: 1.06			
Glycolysis/Gluconeogenesis Microbial metabolism in diverse environments	12 **17**	6-phosphofructokinase (pfkA) MW1642 Acetate--CoA ligase (acsA) MW1676 Bifunctional acetaldehyde-CoA/alcohol dehydrogenase (adhE) MW0123 Glucose-6-phosphate isomerase (pgi) MW0844 Glucokinase (glcK) MW1499 Glyceraldehyde-3-phosphate dehydrogenase (gap) MW0734 Phosphoglycerate kinase (pgk) MW0735 Pyruvate kinase (pykA) MW1641 Triosephosphate isomerase (tpiA) MW0736 PTS system transporter subunit IIA (crr) MW1312 Fructose-1,6-bisphosphate aldolase (fdaB) MW2525 Phosphoglucomutase (pgcA) MW2411–– Glutamate-1-semialdehyde aminotransferase (gsaB) MW1804 Carbamate kinase (arcC) MW2553 Delta-aminolevulinic acid dehydratase (hemB) MW1612 Dipeptidase PepV (pepV) MW1694 Formate--tetrahydrofolate ligase (fhs) MW1675 Fructose-1,6-bisphosphate aldolase (fdaB) MW2525 Fumarate hydratase (fumC) MW1792 3-hexulose-6-phosphate synthase (hxlA) MW0525 Citrate synthase (citZ) MW 1639 Phosphoglucomutase (pgcA) MW2411 Glyoxalase MW2442 Malate:quinone oxidoreductase (mqo1) MW2286 Nitrate reductase subunit beta (narH) MW2318 Putative translaldolase (tal) Mw1721 Respiratory nitrate reductase subunit gamma (narI) MW2316 Sulfite reductase (NADPH) flavoprotein subunit alpha (cysJ) MW2540 Uroporphyrinogen III synthase (hemD) MW1613	3.2 × 10^−2^ 2.3 × 10^−1^
Annotation Cluster 2 Enrichment Score: 0.88			
Mismatch repair Homologous recombination DNA replication	7	ATP-dependent DNA helicase PcrA MW1846 ATP-dependent DNA helicase RecG (recG) MW1110 DNA polymerase III PolC (polC) MW1147 DNA polymerase III subunit delta (holA) MW1538 DNA repair protein RecO (MW1518) DNA polymerase III subunit epsilon MW1835 DNA primase (SAOUHSC_01663) NAD-dependent DNA ligase (lig) MW1845 Single-stranded-DNA-specific exonuclease RecJ MW1586 Recombination and DNA strand exchange inhibitor protein (mutS2) MW1027	4.3 × 10^−2^ 2.1 × 10^−1^ 2.5 × 10^−1^

**Table 7:** Antimicrobial resistance and virulence resistance related DEGs by transcriptomics.

Gene ID	Description	RPKM 1-R	Expression 1-R	RPKM 1-S	Expression 1-S	*q-*Value
** AMR-RELATED TRAITS **						
**Overexpression**						
MW2277	Teicoplanin resistance-associated protein A (TcaA)	33	54	0	0	0
MW0005	DNA gyrase subunit B (GyrB)	42	68	0	0	0
MW2005	UDP-N-acetylmuramoylalanyl-D-glutamyl-2, 6-diaminopimelate- D-alanyl-D-alanyl ligase (MurF)	36	59	0	0	0
MW0814	D-alanine--poly(phosphoribitol) ligase subunit 1 (DltA)	83	135	0	0	0
* MW1247 *	Oxacillin resistance-related FmtC protein (MprF)	23	38	0	0	0
**Underexpression**						
MW0657	Quinolone resistance protein (NorA)	0	0	14	21	0
MW1325	Quinolone resistance protein (NorB)	0	0	18	26	0
MW0111	Tetracycline resistance protein (Tet38)	0	0	93	0	0
** VIRULENCE-RELATED TRAITS **						
*** Adhesion ***						
** Underexpression **						
MW0516	Ser-Asp-rich fibrinogen-binding bone sialoprotein-binding protein SdrC	0	0	20	30	0
MW0517	Ser-Asp-rich fibrinogen-binding bone sialoprotein-binding protein SdrD	0	0	12	18	0
MW1880	Truncated cell surface protein map-w (EapP/Map)	0	0	11	16	0
*** Effector delivery system ***						
** Underexpression **						
MW0259	Type VII secretion protein EsaA	0	0	17	26	0
*** Exotoxins ***						
** Underexpression **						
MW1959	Delta-hemolysin (*hld*)	0	0	1685	2516	0
MW1044	Alpha-hemolysin (*hla)*	0	0	21	31	0
MW2344	Gamma-hemolysin component B (*hlgB*)	0	0	21	31	0
MW2343	Gamma-hemolysin component C (*hlg*C)	0	0	38	57	0
MW0051	Enterotoxin H (*seh*)	0	0	28	42	0
MW1552	Enterotoxin homologous Protein	0	0	36	37500	0
*** Membrane-acting toxin and Superantigen ***						
** Underexpression **						
MW0084	Immunoglobulin G binding protein A precursor (SpA)	0	0	23	34	6.92 × 10^−94^
*** Exoenzyme *** s						
**Underexpression**						
MW2590	Triacylglycerol lipase precursor (Lip)	0	0	7	11	2.97 × 10^−8^
MW1754	Serine protease SplB	0	0	28	42	0
*** Immune modulatory proteins ***						
**Overexpression**						
MW0137	Capsular polysaccharide synthesis enzyme Cap8N	1	27	0	0	0
MW2341	IgG-binding protein SBI (*sbi*)	24	40	0	0	8.22 × 10^−3^
** Underexpression **						
* MW0126 *	Capsular polysaccharide synthesis enzyme Cap8C	0	0	0	20	1.01 × 10^−3^
* MW0130 *	Capsular polysaccharide synthesis enzyme Cap8G	0	0	0	20	1.35 × 10^−3^
MW0139	Capsular polysaccharide synthesis enzyme Cap8P	0	0	14	21	0
MW2559	Immunodominant antigen B (IsaB)	0	0	82	122	0
*** Biofilm ***						
** Underexpression **						
MW2586	Poly-beta-1,6-N-acetyl-D-glucosamine synthase *ica*A	0	0	13	20	0

**AMR AND VIRULENCE TRANSCRIPTIONAL REGULATORS**						
**Overexpression**						
*MW1824*	Two-component response regulator *VraR*	232	378	0	0	0
*MW1825*	Two-component sensor histidine kinase V*ra*S	270	438	0	0	0
* MW1988 *	RNA polymerase sigma factor SigB	2	67	0	4	5.39 × 10^−3^
MW0668	Response regulator SaeR	89	145	0	0	0
MW1705	Repressor toxin Rot	72	117	0	0	1.87 × 10^−3^
** Underexpression **						
MW0018	*Response regulatory protein WalR*	0	0	27	0	0
MW0019	*Sensor protein kinase WalK*	0	0	23	0	0
MW1446	Respiratory response protein SrrA	0	0	139	208	0
MW1960	Accessory gene regulator B (AgrB)	0	7	30	789	2.9 × 10^−53^
MW1962	Accessory gene regulator C (AgrC)	0	0	8	212	0
MW1963	Accessory gene regulator A (AgrA)	0	11	27	717	2.5 × 10^−57^
MW0085	HTH-type transcriptional regulator SarS (SarS)	0	0	26	39	0
MW0621	Response regulator protein GraR	0	0	32	48	0

## Data Availability

Genomic and transcriptomic reads can be found in National Center for Biotechnology Information (NCBI) Genome database in the Sequence Read Archive (SRA) under the BioProject PRJNA860577 with the Biosample Genomic Paired End and RNA-seq raw sequences n° SAMN29849119, SAMN29849120 and with Biosample Genomic mate-pair draft sequences n°SAMN30428603, SAMN30428604.

## Supplementary Materials

The following are available online at https://www.mdpi.com/article/10.3390/antibiotics11091159/s1, Table S1: Total number of paired end and mate pair reads and Estimated Coverage Generated by the Trimming after the sequencing process, Table S2A,B: Summary Table of Post-Assembly Statistics and Metrics generated using the Quast software, Table S3. Primer set used in real time qPCR Validation.
