# Supplementary material for: Balancing the Virulence and Antimicrobial Resistance in VISA DAP-R CA-MRSA Superbug
Source: Antibiotics (Basel). 2022 Aug 27;11(9):1159. doi: 10.3390/antibiotics11091159 (PMC9495084; doi:10.3390/antibiotics11091159)
Supplement: Supplementary file 1 [file antibiotics-11-01159-s001.zip › Table S1.pdf]

Table S1. Total number of paired end and mate pair reads and Estimated Coverage Generated by the Trimming after the sequencing process

Sample	Filtered PE (M)	Filtered MP (M)	E. Coverage
1-S	8,35M (*2)	1,4M (*2)	556,75X
1-R	3,81M (*2)	3,39M (*2)	492,98X
