# Supplementary material for: Balancing the Virulence and Antimicrobial Resistance in VISA DAP-R CA-MRSA Superbug
Source: Antibiotics (Basel). 2022 Aug 27;11(9):1159. doi: 10.3390/antibiotics11091159 (PMC9495084; doi:10.3390/antibiotics11091159)
Supplement: Supplementary file 1 [file antibiotics-11-01159-s001.zip › Table S2.pdf]

TableS2 A-B. Summary Table of Post-Assembly Statistics and Metrics generated using the Quast software

A) 1-S Assembly Report

	scaffolds broken	scaffolds
# contigs (>= 0 bp)	137	122
# contigs (>= 1000 bp)	24	9
Total length (>= 0 bp)	2854642	2857722
Total length (>= 1000 bp)	2827578	2830658
# contigs	27	12
Largest contig	372493	1046768
Total length	2829856	2832936
GC (%)	32.74	32.74
N50	237590	696706
N75	123017	428695
L50	5	2
L75	9	3
# N's per 100 kbp	0.21	108.93

B) 1-R Assembly Report

	scaffolds broken	scaffolds
# contigs (>= 0 bp)	117	112
# contigs (>= 1000 bp)	27	22
Total length (>= 0 bp)	2846168	2846625
Total length (>= 1000 bp)	2824080	2824537
# contigs	32	27
Largest contig	888452	1543272
Total length	2827637	2828094
GC (%)	32.72	32.72
N50	272869	1543272
N75	108221	109923
L50	3	1
L75	8	4
# N's per 100 kbp	0.04	16.19

All statistics are based on contigs of size >= 500 bp, unless otherwise noted (e.g., "# contigs (>= 0 bp)" and "Total length (>= 0 bp)" include all contigs).
