# Supplementary material for: Balancing the Virulence and Antimicrobial Resistance in VISA DAP-R CA-MRSA Superbug
Source: Antibiotics (Basel). 2022 Aug 27;11(9):1159. doi: 10.3390/antibiotics11091159 (PMC9495084; doi:10.3390/antibiotics11091159)
Supplement: Supplementary file 1 [file antibiotics-11-01159-s001.zip › Table S3.pdf]

Table S3. Primer set used in real time qPCR Validation

Primer	Sequences (5'→3')	Fragment Size (bp)
*mur*F-Fw	CGTCACAGGGTCTAATGGTAAA	224
*mur*F-Rw	CAATATCTGGTTGAGCGAGGTT	
hld-Fw	CTGAGTCCAAGGAAACTAACTCTAC	235
hld-Rw	ATCTTGTGCCATTGAAATCA	
hla-Fw	AGGTTCCATATTGATGAATCCTG	321
hla-Rw	GCTACTTCATTATCAGGTAGTTGC	
dltA-Fw	ATGTTTAGCATCAGGCGGTAC	247
dltA-Rw	ACTTGGGAAACGGCTCACTAA	
*mpr*F-Fw	GAACCACCGTTTTCAACTGAA	244
*mpr*F-Rw	GTAAATCTAACTCTGGCAACCATC	
spa-Fw	AGAACAACGCAATGGTTTCA	215
spa-Rw	GATCGTCTTTAAGGCTTTGGAT	
agrA-Fw	TTAACAACTAGCCATAAGGATGTG	310
agrA-Rw	CGTTACGAGTCACAGTGAACTTAC	
icaA-Fw	CATTGAACAAGAAGCCTGACA	301
icaA-Rw	ATATGATTATGTAATGTGCTTGGATG	
sdrD-Fw	AGTACACAGTGGGAACAGCATCA	225
s*drD*-Rw	CATTACCTTGAGATGATACCATTTCT	
gyrB-Fw	CAACTATGAAACATTACAGCAGCGT	256
gyrB-Rw	TGTGGCATATCCTGAGTTATATTGAAT	
